# Supplementary material for: Genome-Wide Identification and Posttranscriptional Regulation Analyses Elucidate Roles of Key Argonautes and Their miRNA Triggers in Regulating Complex Yield Traits in Rapeseed
Source: Int J Mol Sci. 2023 Jan 28;24(3):2543. doi: 10.3390/ijms24032543 (PMC9916703; doi:10.3390/ijms24032543)
Supplement: Supplementary file 1 [file ijms-24-02543-s001.zip › Figures S1 and S2.pdf]

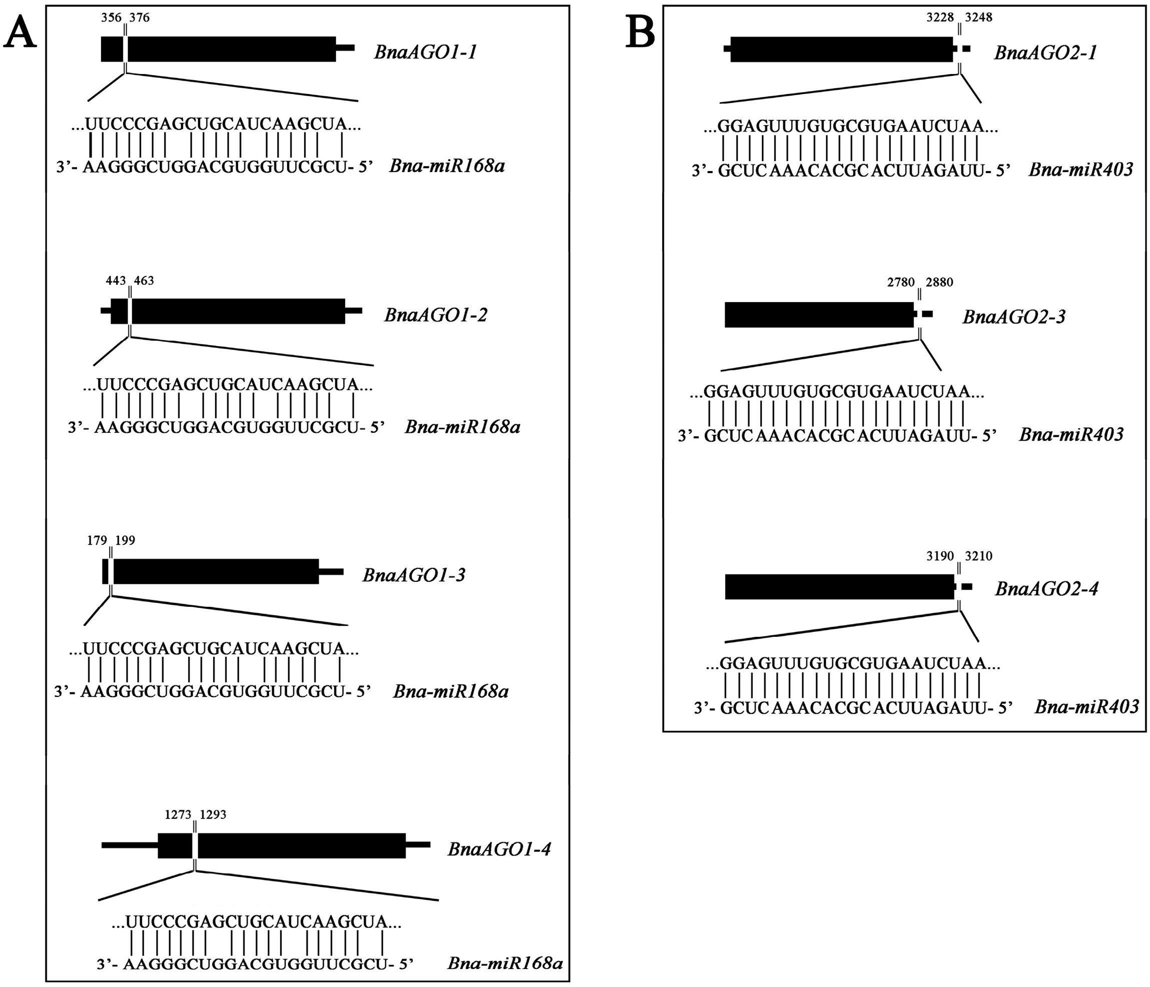

Figure S1: Analysis of the targets set of *B. napus* miR168a and miR403 mediated cleavage of (**A**) BnaAGO1s and (**B**) BnaAGO2s.

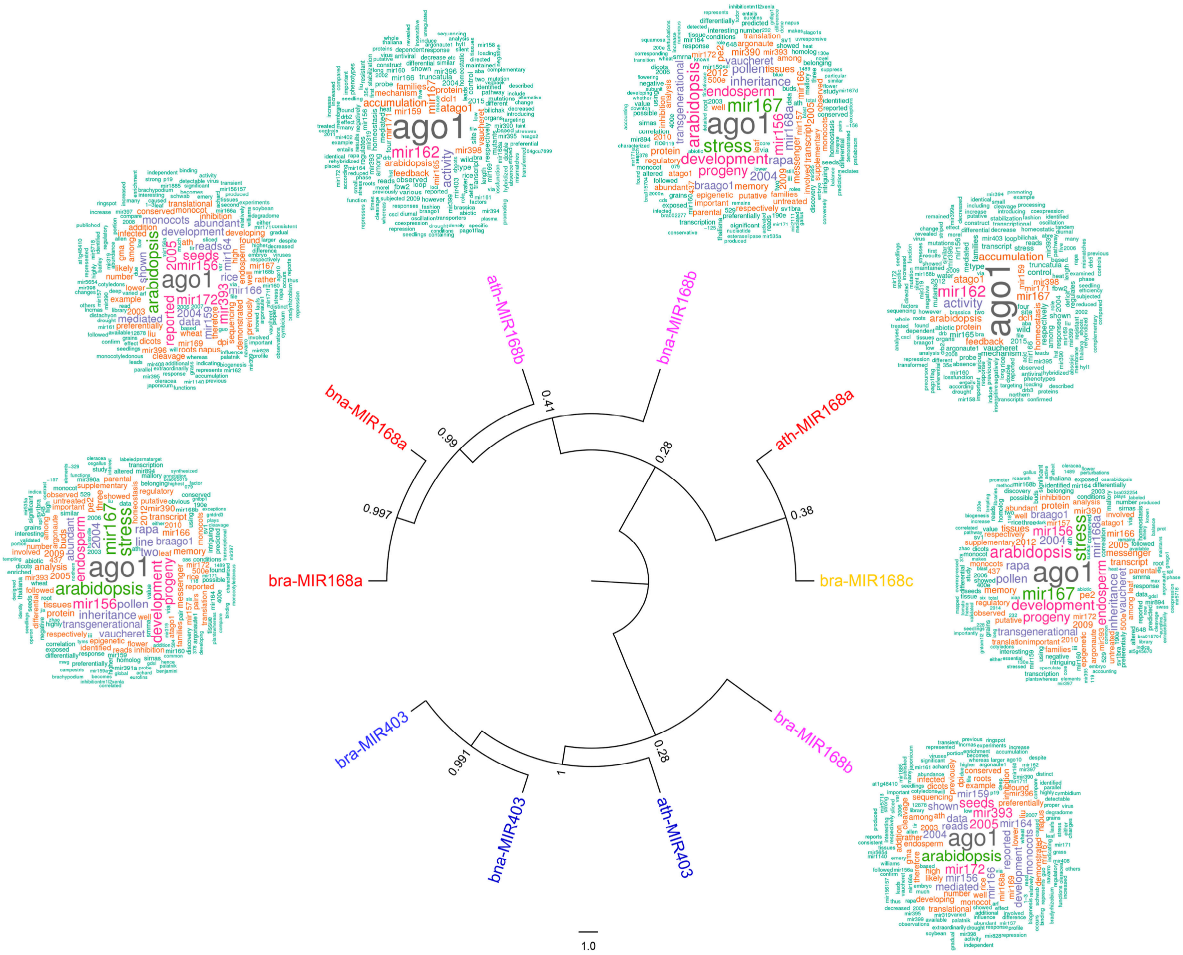

Figure S2: The phylogenetic tree and regulator relationship of miR168 and miR403 among the 4 species.
